# EFFECTS OF COGNITIVE TRAINING ON WHITE MATTER MICROSTRUCTURE AND COGNITION IN OLDER ADULTS: A SYSTEMATIC REVIEW

**DOI:** 10.1093/geroni/igz038.2440

**Published:** 2019-11-08

**Authors:** Grace M McPhee, Luke A Downey, Con Stough

**Affiliations:** Swinburne University of Technology, Melbourne, Victoria, Australia

## Abstract

Adults who remain cognitively active may be protected from age-associated changes in white matter (WM) and cognitive decline. To determine if cognitive activity is a precursor for WM plasticity, the available literature was systematically searched for Region of Interest (ROI) and whole-brain studies assessing the efficacy of cognitive training (CT) on WM microstructure using Diffusion Tensor Imaging (DTI) in healthy adults (> 40 years). Seven studies were identified and included in this review. Results suggest there are beneficial effects to WM microstructure after CT in frontal and medial brain regions, with some studies showing improved performance in cognitive outcomes. Benefits of CT were shown to be protective against age-related WM microstructure decline by either maintaining or improving WM after training. These results have implications for determining the capacity for training-dependent WM plasticity in older adults and whether CT can be utilised to prevent age-associated cognitive decline. Additional studies with standardised training and imaging protocols are needed to confirm these outcomes.

